# Iron in intracellular infection: to provide or to deprive?

**DOI:** 10.3389/fcimb.2013.00096

**Published:** 2013-12-09

**Authors:** Sandro Silva-Gomes, Sílvia Vale-Costa, Rui Appelberg, Maria S. Gomes

**Affiliations:** ^1^Infection and Immunity Unit, Instituto de Biologia Molecular e Celular, Universidade do PortoPorto, Portugal; ^2^Department of Molecular Biology, Instituto de Ciências Biomédicas Abel Salazar, Universidade do PortoPorto, Portugal

**Keywords:** transition metal, immunity, *Mycobacterium*, *Leishmania*, infection

## Abstract

Due to their chemical versatility, transition metals were incorporated as cofactors for several basic metabolic pathways in living organisms. This same characteristic makes them potentially harmful, since they can be engaged in deleterious reactions like Fenton chemistry. As such, organisms have evolved highly specialized mechanisms to supply their own metal needs while keeping their toxic potential in check. This dual character comes into play in host-pathogen interactions, given that the host can either deprive the pathogen of these key nutrients or exploit them to induce toxicity toward the invading agent. Iron stands as the prototypic example of how a metal can be used to limit the growth of pathogens by nutrient deprivation, a mechanism widely studied in *Mycobacterium* infections. However, the host can also take advantage of iron-induced toxicity to control pathogen proliferation, as observed in infections caused by *Leishmania*. Whether we may harness either of the two pathways for therapeutical purposes is still ill-defined. In this review, we discuss how modulation of the host iron availability impacts the course of infections, focusing on those caused by two relevant intracellular pathogens, *Mycobacterium* and *Leishmania*.

## Iron in biologic systems and infection processes

Transition metals are elements that have an incomplete inner electron shell and can easily shift between different oxidation states. Cells took advantage of this property and included transition metals into proteins, gaining catalytic potential and protein stability. The function of transition metals in enzymatic catalysis can be divided into two groups, depending upon the metal acting as a redox center or not. Of the redox active metals, iron is the most prevalent, followed by copper and molybdenum, while zinc is the most common non-redox transition metal (Andreini et al., 2008). Iron is found in an unparalleled variety of sites and cofactors, such as haem groups and iron-sulphur clusters, and is involved in processes such as oxygen sensing and transport, energy metabolism and nucleic acid synthesis. The predominance of iron is presumably due to the large availability of water soluble ferrous iron during prebiotic times, before the rise of atmospheric oxygen levels caused by photosynthesis resulted in the precipitation of insoluble iron (III) (Crichton and Pierre, 2001). As a result, almost all living organisms from archaea to eukaryotes require iron in their metabolism. *Borrelia burgdorferi* is unique among pathogens in that it bypassed iron dependence by substituting zinc or manganese for iron in its metalloproteins (Posey and Gherardini, 2000; Nguyen et al., 2007).

### Iron metabolism in the host

Vertebrates have evolved a complex network of proteins to acquire, transport and store iron, while maintaining free iron concentration at very low levels (reviewed in Kaplan and Ward, 2013). In mammals there is no regulated excretion of iron and the replenishment of losses from desquamation and minor bleeding, which account for the loss of less than 0.05% of body iron per day, occurs at the level of intestinal absorption. Dietary iron, either inorganic or bound to haem, is absorbed at the brush border of enterocytes lining the proximal portion of the duodenum (Andrews and Schmidt, 2007). Iron is transported as ferrous iron into circulation through ferroportin, the only cellular iron exporter. Transferrin is the physiological carrier of iron in plasma, binding two atoms of ferric iron. In humans, the normal saturation of transferrin is only 20–40% (Ganz and Nemeth, 2012b). Iron-loaded transferrin binds with high affinity to the transferrin receptor (TFR)-1, ubiquitously expressed at cell surfaces, and is internalized by clathrin-dependent endocytosis. Iron is then released intracellularly and becomes part of the labile iron pool (Correnti and Strong, 2012). Iron that is not needed for immediate use or export is stored in ferritin. In vertebrates, cytosolic ferritin is formed by the spontaneous assembly of 24 subunits of Heavy (H) and Light (L) chains, resulting in a hollow shell capable of accommodating up to 4500 iron atoms in an inert, non-toxic form (Harrison and Arosio, 1996). Erythropoiesis is the most avid consumer of iron in the mammal organism. Approximately 60–70% of the human adult body iron is bound within haemoglobin (~2.5 g). With the erythrocyte lifespan of 120 days, the reutilization of iron recycled from senescent cells accounts for most of the iron flux (Ganz and Nemeth, 2012b). The hormone hepcidin is regarded as the central regulator of systemic iron homeostasis. Hepcidin is a 25 amino acid peptide that is mainly produced by hepatocytes. It binds to the iron exporter ferroportin, causing its internalization and degradation. Consequently, it reduces the absorption of dietary iron by enterocytes and the release of iron from intracellular stores (Ganz and Nemeth, 2012a).

As described, macrophages play a central role in regulating iron metabolism since they recycle haem iron and regulate its storage. However, since macrophages respond to other environmental cues such as signals from the immune system, they may undergo major and distinct physiological adaptations in different settings namely those that are triggered by infection. For example, it is known that classic activation of macrophages (M1 macrophages) e.g., by gamma interferon together with microbial molecules acting as Toll-like receptor ligands modulates a different program of iron handling as compared to the alternative activation (M2 macrophages) induced by cytokines such as interleukin (IL)-4 and IL-13 induced in certain types of infection or by cues from tissues in steady-state conditions (Recalcati et al., 2012). The ensuing consequences at the systemic level are distinct as classic activation promotes the sequestration of iron leading to systemic lowering of its availability whereas alternative activation, by promoting export of the metal by macrophages would lead to the opposite systemic effects.

### Transition metals during infection

**Figure 1:**
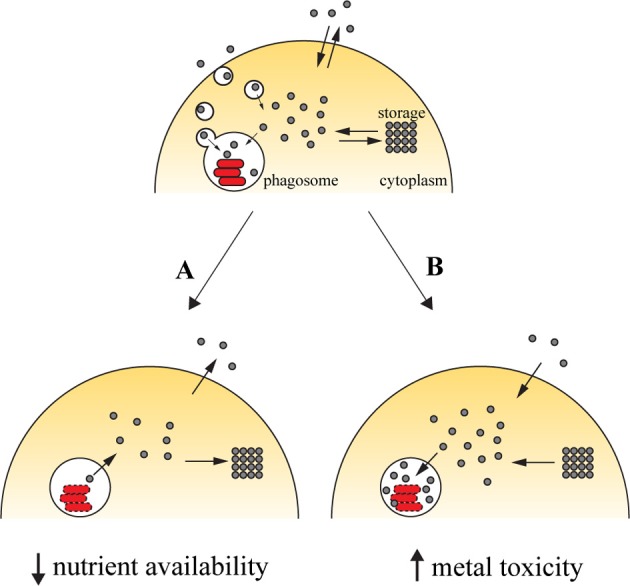
**Transition metals in immunity against intracellular pathogens**. Upon entry into the host cell, the pathogen is able to retrieve metals from its surroundings to incorporate them in mettaloproteins strictly required for its survival. The pathogen uses dedicated transporters and specialized proteins [e.g., siderophores (Hider and Kong, 2010), high-affinity iron chelating compounds] to acquire metals from the cytoplasmic pools or by hijacking the cell pathways of metal acquisition (for example, *Mycobacterium* and *Leishmania* have access to transferrin-bound iron in the endocytic pathway). One of the countermeasures employed by the host cell to reduce the proliferation of the pathogen is metal deprivation **(A)**. This is achieved by pumping out the metal from the phagosome, mediated by metal transporters such as SLC11A1, which is recruited to the phagosomal membrane, where it transports iron and manganese out of this compartment. The metal can then be diverted into storage [e.g., the iron-storage ferritin is induced during infection with *Salmonella* (Nairz et al., 2007) and *Mycobacterium* (Silva-Gomes et al., 2013b)] or exported [e.g., ferroportin, an iron exporter that localizes to the cellular membrane, is induced in macrophages infected with *Mycobacterium* (Van Zandt et al., 2008) and *Salmonella* (Nairz et al., 2007)]. On the other hand, the host cell can explore the toxicity of transition metal and direct them to the microbial invader **(B)**. During infection, metal transporters such as copper transport 1 (CTR1) (White et al., 2009) are induced, and transport the metal from the extracellular environment. Metals are also mobilized from intracellular storage [e.g., macrophages mobilize zinc from intracellular stores when infected with *M. tuberculosis* or *E. coli* (Botella et al., 2011)], which will then accumulate in the phagosome by the action of metal transporters, such as the copper transporter ATP7A (White et al., 2009).

Successful colonization of a host by pathogens requires that these must gain access to the required amounts of transition metals. Vertebrate hosts exploit this requirement by sequestering these elements (Figure 1A). Iron withholding is the most well-studied example of metal deprivation. A complex network of host proteins renders this valuable nutrient largely inaccessible to pathogens, a concept usually known as nutritional immunity (Appelberg, 2006a; Hood and Skaar, 2012). However, recent evidences suggest that this mechanism is also used to sequester other transition metals, including manganese and zinc (Kehl-Fie and Skaar, 2010). The strict requirement for transition metals is due to their involvement in numerous processes ranging from microbial metabolism to accessory virulence factor function. Hence, without the required concentration of these nutrients, the invading agent is unable to proliferate and cause disease (Kehl-Fie and Skaar, 2010).

The host can capitalize on the toxicity of transition metals and increase their concentration in the compartment where pathogens proliferate (Figure 1B). By this mechanism, transition metals can potentiate pathogen killing together with the respiratory burst in phagocytes. The moderately reactive superoxide radical is rapidly reduced to hydrogen peroxide, either spontaneously or enzymatically. In turn, hydrogen peroxide may give rise to the highly reactive and short-lived hydroxyl radicals in the Fenton reaction, by reacting with a reduced transition metal, such as Fe(II) or Cu(I) (Galaris and Pantopoulos, 2008; Hodgkinson and Petris, 2012), resulting in oxidative damage to the pathogen. The toxicity of copper against *M. tuberculosis* has been reported (Wolschendorf et al., 2011) and a copper-transporting ATPase has been identified in macrophages (White et al., 2009). Other mechanisms of metal intoxication independent of oxidative stress have also been proposed. For example, it was suggested that metal cofactor replacement may mediate copper toxicity against bacteria (Rowland and Niederweis, 2012) and that perturbation of a defined ratio of transition metals is detrimental to bacterial physiology, which may underlie zinc and manganese toxicity (Botella et al., 2011; Hood and Skaar, 2012).

This review focuses on the effect of availability of iron, the most abundant transition metal in the vertebrate host, during infection with two intracellular pathogens, *Mycobacterium* and *Leishmania*.

## Iron metabolism in *Mycobacterium* infection

Tuberculosis remains the most important bacterial infection worldwide. It is estimated that one third of the world population is infected with *M. tuberculosis*. However, in 90% of the cases immunity is able to prevent disease, leading in the majority of cases to latent infection. The advent of the AIDS epidemic and the introduction of immunosuppressive therapies dramatically increased the number of people at risk of infection not only with *M. tuberculosis* but also with other *Mycobacterium* species that otherwise would not cause disease. This is the case of *Mycobacterium avium*, a Non Tuberculous Mycobacteria (NTM). Whereas *M. tuberculosis* is a primary pathogen, *M. avium* is seldom identified as one. The two mycobacterial species differ in that opportunistic infection by *M. avium* occurs in advanced stages of AIDS when blood CD4^+^ T cell counts are lower than 50 per mm3, whereas infection of AIDS patients with *M. tuberculosis* is not limited to such late stages of the disease. *M. tuberculosis* has no known reservoir other than humans. The bacteria are almost exclusively transmitted by aerosolized droplets, generated by the cough or sneeze of a person with *M. tuberculosis* lung infection and are inhaled by an uninfected person. In contrast, *M. avium* does not seem to be transmitted between hosts and infection occurs usually through tap water. Although mycobacteria can infect several cell types (e.g., epithelial cells, eosinophils, neutrophils and dendritic cells) the macrophage has long been established as the central cell during the infection, being the primary player of cellular immunity as well as the main site of bacterial replication. Despite the differences that make *M. tuberculosis* a highly successful pathogen and *M. avium* an opportunistic infectious agent that mostly affects patients with compromised immunity, studies in the mouse model showed that immune response to both mycobacteria is similar, namely regarding the pivotal roles of CD4^+^ T cells, macrophages, and the IL12–IFNγ cytokine axis. This is also the case of iron availability, as it will be discussed below.

For a review regarding pathogenesis of infection with *M. tuberculosis* and *M. avium* we refer the readers to (Cooper, 2009) and (Appelberg, 2006b), respectively.

### Host iron state: evidences from human studies

Iron availability can be a determinant factor during infections with mycobacteria. In the nineteenth century, Armand Trousseau, a French physician, already documented the dangers of giving iron to patients suffering from tuberculosis. He observed that although tuberculosis patients developed some degree of anaemia, iron supplementation in their diet resulted in a poorer outcome than those patients who did not receive additional iron (Trousseau, 1872). Contemporary studies have corroborated this link between iron and tuberculosis. In 1996, Gordeuk et al. reanalyzed an autopsy study performed in South Africa in the 1920s, and found a relationship between death from tuberculosis and hepatic and splenic iron overload, notably with increased iron deposition in the mononuclear macrophage system (Gordeuk et al., 1996). Epidemiological studies in rural Zimbabwe have also shown a correlation between iron overload induced by dietary intake of iron (although genetic predisposition could also be a factor), and the risk of active pulmonary tuberculosis (Gangaidzo et al., 2001). Finally, a recent study has shown iron redistribution to macrophages in HIV infected patients to be a risk factor for the development of tuberculosis (McDermid et al., 2013).

Owing to the strict regulation of intestinal absorption, iron overload of mammalian organisms is rare. However, certain genetic diseases can lead to iron overload, such as hereditary hemochromatosis (HH), the most common inherited single gene disorder in people of Northern and Western Europe. This disease is most often associated with mutations in a molecule, HFE, homologous to class I major histocompatibility complex (MHC) alpha chains. These patients spontaneously develop hepatic iron overload, with iron accumulation in hepatocytes but not in macrophages. Given the reduced capacity of the macrophage to retain iron, human macrophages from HH patients where shown to be less permissive to *M. tuberculosis* growth (Olakanmi et al., 2007). However, whether this is true at an all-organism level is not known, as epidemiologic data from the incidence of tuberculosis in HH patients are not available. Furthermore, results from mouse models of HH indicated that the overall tissue iron overload contributes to susceptibility to mycobacteria (discussed below).

### Host iron state: evidences from the mouse model

For the most part, the findings in humans described above have been very effectively reproduced in the mouse model of experimental infections with mycobacteria.

Our group has shown that an iron poor diet led to a reduced proliferation of *M. avium* in mice (Gomes et al., 1999a), while the parenteral administration of iron-dextran led to an increased proliferation of the bacilli (Gomes and Appelberg, 1998; Gomes et al., 2001). Likewise, others have shown that either the parenteral overload of iron in the form of polymaltose ferric hydroxide (Lounis et al., 1999, 2001) (Lounis et al., 2003) or the administration of iron citrate through the drinking water (Schaible et al., 2002) rendered the mice more susceptible to experimental infection with *M. tuberculosis*.

Mice deficient in HFE or in the HFE-binding protein beta-2-microglobulin (β 2m^−/−^) spontaneously develop hepatic iron overload, similar to HH patients (De Sousa et al., 1994; Zhou et al., 1998). β 2m^−/−^ mice are known for a long time to be more susceptible to *M. tuberculosis*, an effect initially attributed to a lack of major histocompatibility complex class I (MHC-I) restricted cells (Flynn et al., 1992). However, MHC-I-KO animals are less susceptible to *M. tuberculosis* than β 2m^−/−^ mice (Rolph et al., 2001; Schaible et al., 2002). Schaible et al. have shown that correcting the iron overload in these mice, through the administration of lactoferrin, reduced their mycobacterial loads to levels comparable to MHC-I-KO animals, proving that iron availability is the major factor accounting for the increased susceptibility of β 2m^−/−^ mice to experimental tuberculosis (Schaible et al., 2002). In our group, we also found that the increased accumulation of iron in β 2m^−/−^ mice is associated with an increased susceptibility to *M. avium* experimental infection (Gomes-Pereira et al., 2008). Furthermore, we showed a similar effect in HFE^−/−^ mice (Gomes-Pereira et al., 2008). Interestingly, although these models of iron overload tend to accumulate iron in hepatocytes and not in macrophages, we observed that during infection iron accumulates in macrophages within granulomas (Gomes-Pereira et al., 2008).

### The fight for iron

In order to successfully establish an infection, mycobacteria must gain access to iron and have therefore evolved strategies to acquire iron from the host. Mycobacteria are able to block phagosomal maturation, replicating in an intracellular compartment with access to iron-loaded transferrin (Clemens and Horwitz, 1996; Halaas et al., 2010). Interestingly, the ability of *M. avium* to prevent phagosome maturation was shown to be dependent on its capacity to acquire iron (Kelley and Schorey, 2003). In addition to transferrin, *M. tuberculosis* was also shown to be able to acquire iron from lactoferrin (Olakanmi et al., 2004) and from the macrophage cytoplasmic iron pools (Olakanmi et al., 2002). Furthermore, *M. tuberculosis* can use haem as an iron source (Jones and Niederweis, 2011; Tullius et al., 2011; Nambu et al., 2013). In line with the notion that access to iron is a requisite to cause a persistent infection, it was reported, using X-ray microscopy, that iron concentration increased over time in the phagosomes of macrophages infected with *M. avium* or *M. tuberculosis*, while it decreased in those infected with the non-pathogenic *M. smegmatis* (Wagner et al., 2005). Several bacteria are able to produce siderophores, low molecular weight molecules with high affinity for iron. Pathogenic mycobacteria, such as *M. tuberculosis* and *M. avium*, synthetize siderophores that remain associated to the cell wall, termed mycobactins, and others that are secreted, named carboxymycobactins (Ratledge, 2004). These siderophores are able to remove iron from host iron-binding proteins, such as transferrin and lactoferrin (Gobin and Horwitz, 1996). In *M. tuberculosis* a gene cluster that encompasses 10 genes, designated mbtA-J, encodes the machinery responsible for siderophore biogenesis (Quadri et al., 1998). *mbtB* (De Voss et al., 2000), *mbtD* (Jones and Niederweis, 2011), and *mbtE* (Reddy et al., 2013) mutants show restricted growth in iron-deficient medium and in macrophages, suggesting that siderophore synthesis is required for *M. tuberculosis* virulence. Indeed, guinea pigs infected with the *mtbE* mutant exhibited reduced bacillary load in comparison with animals infected with the parental strain (Reddy et al., 2013). However, these latter data should be confirmed since this study showed that the mutant strain induced the same amount of moderate pathology in the lung as the wild type strain despite its virtually complete elimination by 4 weeks of infection. Whether that was due to the high inoculum used and the persistence of inflammatory material from the mycobacteria or to an underestimation of the bacterial loads given the difficulties in growing the mutant strain *in vitro* is unclear. Noteworthy, whereas pathology subsequently exacerbated in the hosts infected with the wild type strain, it did not in animals infected with the mutant strain. In addition to the synthesis of siderophores, proteins involved in their import or export are also critical for virulence. IrtAB, a transporter of iron-loaded carboxymycobactin, is required for the replication of *M. tuberculosis* in macrophages and *in vivo* in a mouse model of aerosol infection (Rodriguez and Smith, 2006). A siderophore export system was recently identified in *M. tuberculosis*, involving the proteins MmpS4 and Mmsp5 (Wells et al., 2013). Deletion of both *mmpS4* and *mmpS5* drastically decreases synthesis and secretion of siderophores and notably reduces *M. tuberculosis* virulence in mice (Wells et al., 2013).

The essentiality of iron acquisition systems for mycobacterial survival has led to the suggestion that this pathway could constitute a new drug target in anti-mycobacteria drug development (Appelberg, 2006a; Meyer, 2006). In fact, several compounds with the capacity to interfere with siderophore synthesis and/or function were shown to have strong inhibitory activity against *M. tuberculosis* (Ferreras et al., 2005, 2011). It should be noted that although some compounds that interfere with siderophore synthesis showed promising results in axenic cultures of *M. tuberculosis* (Ferreras et al., 2005), their effect in a mouse model of *M. tuberculosis* infection is very limited (Lun et al., 2013). These observations may indicate that (1) mycobacteria have access *in vivo* to others forms of iron independent of siderophore acquisition [e.g., haem iron (Jones and Niederweis, 2011)] or (2) the compound is not reaching the cells harboring mycobacteria. Alternative possible targets for new drug development may instead include the siderophore transport systems. We have also shown that the addition of iron chelators to *M. avium* cultures, either in axenic culture, in macrophage cultures, or *in vivo* led to significant decreases in mycobacterial growth (Gomes et al., 1999a; Fernandes et al., 2010). Furthermore, we have developed new molecules based on the 3-hydroxy-4-pyridinone iron chelating moiety, in which the inclusion of a rhodamine residue improved anti-mycobacterial activity, presumably through improved intracellular distribution and targeting for the mycobacteria-containing phagosome (Fernandes et al., 2010; Nunes et al., 2010; Moniz et al., 2013).

During an infection, the host uses several mechanisms to withhold iron access from pathogens. Anaemia often develops during acute or chronic infections, which is known as anaemia of chronic disease (ACD), and is thought to represent a mechanism to limit iron availability to the invading microorganisms (Weiss, 2009). ACD, the second most common type of anaemia (after anaemia of iron-deficiency) is characterized by hypoferremia (low serum iron) and increased iron retention within the mononuclear phagocyte system (Roy, 2010). Anaemia was described in the mouse model of infection with *M. avium* (Rodrigues et al., 2011) and *M. bovis* BCG (Marchal and Milon, 1981), and epidemiologic studies showed that tuberculosis is frequently associated with anaemia (Lee et al., 2006; Sahiratmadja et al., 2007). One of the putative links between immunity and the anaemia is hepcidin, a peptide that regulates iron homeostasis by mediating the degradation of ferroportin, an iron exporter protein. *In vitro*, infection of macrophages with *M. avium* and *M. tuberculosis* induce the hepcidin mRNA expression (Sow et al., 2007). However, using a microarray platform to analyse the iron-related genes regulated by *M. avium* infection, we did not find hepcidin to be induced in the liver (Rodrigues et al., 2011), questioning the role of hepcidin in the development of anaemia during mycobacterial infections. Nevertheless, hepcidin produced by infected macrophages may have a local effect, rather than a major role in the systemic iron regulation. Indeed, Sow et al. (2007) showed that hepcidin localizes to the mycobacteria-containing phagosomes and possesses direct antimicrobial activity against *M. tuberculosis*, causing structural damage to the mycobacteria. How anaemia develops in the context of mycobacterial infection remains to be determined. Anaemia independent of the expression of hepcidin has been observed in other situations, namely after LPS administration to TIR domain-containing adaptor protein (TRIF)-deficient mice (Layoun et al., 2012) and Hepcidin^+/−^ mice (Deschemin and Vaulont, 2013), in a murine model of protracted peritonitis (Schubert et al., 2012) and notably following TNF administration to mice (Laftah et al., 2006).

There is experimental evidence that some degree of haemolysis and release of free haem may occur during severe infection (Larsen et al., 2010). Free haem contributes to an increased susceptibility to mycobacterial infection, most likely involving mechanisms that differ from those associated to other forms of iron, and haem-oxygenase-1, the enzyme responsible for haem detoxification is essential for host protection against these infections (Silva-Gomes et al., 2013a).

One of the ways by which the host can interfere with the pathogen acquisition of iron is through the action of lipocalin (Lcn)-2. Lcn-2, also known as siderocalin, neutrophil gelatinase-associated lipocalin (NGAL) or 24p3, is capable of binding siderophores (Goetz et al., 2002) and transport them into cells by endocytosis, after interacting with a specific cell-surface receptor (Devireddy et al., 2005). Lcn-2 is able to bind carboxymycobactin from mycobacteria (Holmes et al., 2005) and inhibit the growth of *M. tuberculosis* and *M. bovis* BCG in liquid culture, in a dose-dependent manner reversible by the addition of iron or recombinant carboxymycobactin (Saiga et al., 2008). Furthermore, Lcn-2-deficient mice are highly susceptible to intratracheal infection with *M. tuberculosis* (Saiga et al., 2008). Of note, recent studies have shown that Lcn-2 can also function as an iron shuttle in basal metabolism of the host, either delivering iron to or stealing iron from specific cell types, implicating Lcn-2 in processes unrelated to its function in immunity (Correnti and Strong, 2012).

Inside infected macrophages, pathogen's access to iron may be limited by SLC11A1. SLC11A1 is a divalent metal transporter, recruited to the late endosomal and phagosomal membrane of macrophages and other professional phagocytes (Nevo and Nelson, 2006). Although SLC11A1 contributes to macrophages' efficiency in the recycling of erythrocyte-derived iron (Soe-Lin et al., 2009), the main function of SLC11A1 seems to be the protection against microbes. The *Slc11a1* gene is present in inbred strains of mice in two allelic forms which determine the resistance or susceptibility to several intracellular pathogens such as *Mycobacterium* spp., *Salmonella* spp and *Leishmania* spp (Vidal et al., 1993). Susceptible mice carry a glycine (G) to aspartic acid (D) substitution at position 169, resulting in a misfolding and loss of function of the protein (White et al., 2004). This mutation confers susceptibility to several mycobacterial species, such as *M. avium* (Appelberg and Sarmento, 1990; Gomes and Appelberg, 1998), *M. intracellulare* (Goto et al., 1989), *M. bovis* BCG (Gros et al., 1981) but not to *M. tuberculosis* (Medina and North, 1996; North et al., 1999). Although the G169D mutation has never been found in humans, polymorphic variations at or near human *SLC11A1* are associated with susceptibility to tuberculosis and leprosy in populations from areas of endemic disease (Fortier et al., 2005). The directionality of metal transport in the SLC11A1 is still not consensual. Some groups suggest that iron is transported via this protein into the pathogen-containing phagosome, causing the death of the pathogen by catalyzing the formation of reactive oxygen species (ROS) (Kuhn et al., 1999, 2001; Zwilling et al., 1999), while others argue for an iron efflux from the phagosome, restricting pathogen growth by iron deprivation (Gomes and Appelberg, 1998; Mulero et al., 2002). Studies of analogy with DMT1 (NRAMP2) (Forbes and Gros, 2003), the resistance of mycobacteria to ROS (Segal et al., 1999; Gomes and Appelberg, 2002) and the role of SLC11A1 in erythrocyte recycling (Soe-Lin et al., 2009) favor the second hypothesis, which is now seen as more consensual in the literature (Schaible and Kaufmann, 2004; Cellier et al., 2007; Wessling-Resnick, 2010).

Another iron transporter expressed in macrophages is ferroportin (SLC40A1). Ferroportin is present in the macrophage cytoplasmic membrane and is responsible for iron export (Knutson et al., 2005). Overexpression of ferroportin was reported to inhibit the intra-macrophagic growth of *M. tuberculosis* presumably through iron deprivation (Johnson et al., 2010). An interplay between the expression of ferroportin and the activation of the macrophage's inducible nitric oxide synthase (NOS2) seems to occur. While the overexpression of ferroportin decreased macrophages nitric oxide production (Johnson et al., 2010), the expression of NOS2 was reported by Nairz et al to be necessary to maintain ferroportin expression and iron efflux (Nairz et al., 2013). In this study, *Salmonella* residing within nitric oxide synthase deficient (*Nos2*^−/−^) macrophages acquired more iron than bacteria within wild-type macrophages and the authors concluded that iron deprivation is the main way through which NOS2 contributes to *Salmonella* control. However, while *M. avium* depends on access to iron to proliferate inside macrophages, *Nos2*^−/−^ macrophages and mice are not more permissive to the growth of this mycobacterium (Gomes et al., 1999b), suggesting that the mechanism for the regulation of iron availability by NO during *Salmonella* infection does not apply to *M. avium* infections.

## Iron metabolism in *Leishmania* infection

The leishmaniases are a complex of mammalian diseases characterized by distinct clinical manifestations: cutaneous, mucocutaneous and visceral leishmaniasis. Though considered to be neglected tropical diseases, their global incidence is a worrisome 2 million new cases per year. They are caused by protozoa of the genus *Leishmania*, whose transmission occurs through the bite of female sandfly. Transmission mediated by the insect may be zoonotic—between reservoir hosts (rodents and canines) and humans—or anthroponotic (Ready, 2010). Disease is not necessarily the final outcome in endemic areas where humans stably share the habitat with wild rodents, dogs and the sand fly populations as asymptomatic infections may occur.

*Leishmania* parasites have a digenetic life cycle, alternating between the promastigote stage in the insect gut and the amastigote stage in macrophages of mammalian hosts. Although *Leishmania* can infect diverse host cells (e.g., dendritic cells and neutrophils), there is only evidence for replication and long-term survival within mononuclear phagocytes (Kaye and Scott, 2011).

### Host iron state: evidences from human and canine studies

A direct link between host iron status and human or canine susceptibility to *Leishmania* infection is not clearly found from available epidemiologic data. The fact that several studies found an association between susceptibility to leishmaniasis and polymorphisms in the *SLC11A1* gene, which codes for a transmembrane transporter of iron and other divalent metals, as mentioned before (Altet et al., 2002; Bucheton et al., 2003; Sanchez-Robert et al., 2008; Blackwell et al., 2009; Castellucci et al., 2010) and, on the other hand, the generalized association between leishmaniasis and malnutrition (Maciel et al., 2008; McCall et al., 2013) are indirect evidences that the host iron status may influence the outcome of the *Leishmania* infection. However, more direct correlations can only be made based on the studies performed in several infection models.

### Host iron state: evidences from rodent models

Despite the lack of direct clinical evidences, we could expect iron to favor the growth of *Leishmania*, similarly to what is found for other pathogens (Weinberg, 2009). The fact that these parasites are equipped with diverse iron acquisition mechanisms and are capable of utilizing various iron sources suggested that iron acquisition was essential for pathogenicity and that iron deprivation could be an effective strategy to control leishmanial infections (Sutak et al., 2008; Taylor and Kelly, 2010). Such hypothesis is supported by the finding that the iron chelators desferrioxamine (DFO) and hydroxypyridin-4-ones moderately inhibit the multiplication of *L. major* and *L. infantum* promastigotes in culture medium (Soteriadou et al., 1995). However, DFO has shown either no effect (Murray et al., 1991) or an inhibitory effect on the intramacrophagic growth of *L. donovani* (Segovia et al., 1989; Das et al., 2009) and *L. amazonensis* (Borges et al., 1998). Moreover, treatment of mice with DFO does not affect the development of skin lesions caused by *L. major* (Bisti et al., 2000), but reduces the hepatic and splenic growth of *L. infantum* (Malafaia et al., 2011). Conversely, feeding mice with an iron-deficient diet did not influence *L. infantum* proliferation (Vale-Costa et al., 2013). Overall, the existing studies do not thoroughly support the notion that iron depletion contributes to the control of leishmanial infections.

Data obtained *in cellulo* concerning the impact of iron supplementation is also not consensual. Iron treatment either favored the intramacrophagic growth of *L. donovani* (Das et al., 2009) and *L. amazonensis* (Borges et al., 1998) and reversed the capacity of activated macrophages to eliminate *L. enriettii* (Mauel et al., 1991) or had no influence on both effects (Murray et al., 1991). Interestingly, the role of this nutrient on the *in vivo* growth of the parasite seems to be dependent on the host species. Iron given to hamsters prophylactically or therapeutically enhanced *L. donovani* replication (Garg et al., 2004). By contrast, *in vivo* iron administration to susceptible mice clearly leads to containment of *L. major* in the skin (Bisti et al., 2000, 2006; Bisti and Soteriadou, 2006) and *L. infantum* in the liver and spleen (Vale-Costa et al., 2013). The anti-leishmanial effect of iron is most likely due to its synergistic interaction with reactive oxygen and nitrogen species produced by the host's professional phagocytes (Bisti et al., 2006; Vale-Costa et al., 2013). Furthermore, the control of *L. major* infection, but not that of *L. infantum*, by iron also correlates with the development of a T helper 1 (Th1)-type immune response (Bisti et al., 2000), which is characterized by (i) an increased ability of splenic cells to present *L. major*-derived peptides, (ii) increased levels of IFNγ and NOS2 and decreased levels of IL-4 and IL-10 transcripts at the lesion site and (iii) reduced levels of serum immunoglobulin (Ig) E and IgG1 and increased levels of IgG2a (Bisti et al., 2000). Notably, iron overloaded mice are also resistant to re-infection with *L. major* (Bisti and Soteriadou, 2006). The iron-induced oxidative burst elicited during both primary and secondary infections with *L. major* is linked to the activation of the transcription factor NF-κB and with an enhanced proliferation of IFNγ-secreting CD4^+^ T cells in the draining lymph nodes (Bisti and Soteriadou, 2006). This is substantiated by the fact that iron and reactive oxygen and nitrogen species can modulate the activation of macrophagic NF-κB signaling pathways (Xiong et al., 2003; Leonard et al., 2004; Galaris and Pantopoulos, 2008) which are known to regulate numerous genes involved in immune and inflammatory responses (Bonizzi and Karin, 2004). Indeed, NF-κB regulates the development of IFNγ-secreting CD4^+^ T cells and concomitant resistance to *L. major* (Artis et al., 2003). Hence, iron not only seems to synergize with the host's oxidative mechanisms of defense, but also interacts with reactive oxygen and nitrogen species in order to activate signaling cascades that regulate the development of protective immunity against *Leishmania*.

The abovementioned reports clearly indicate that iron induces host protection against *Leishmania* infection. Future research should seek a confirmation of the inhibitory effect of iron on the *in vivo* growth of other *Leishmania* species and a better understanding of the molecular pathways involved in the iron-induced resistance against these protozoa.

### The fight for iron

Like many other intracellular pathogens, *Leishmania* must be capable of acquiring iron from the host milieu in order to thrive. Besides holotransferrin (Borges et al., 1998), the growth and survival of *L. infantum* and *L. amazonensis* amastigotes can be supported by iron derived from haemoglobin and hemin (Carvalho et al., 2009). The uptake of haem by intramacrophagic *L. amazonensis* amastigotes is mediated by *Leishmania* haem response 1 (LHR1) protein (Huynh et al., 2012). Furthermore, intracellular *L. amazonensis* also possesses a ferric reductase, the *Leishmania* ferric iron reductase 1 (LFR1) (Flannery et al., 2011) which provides soluble ferrous iron for transport across the parasite plasma membrane by the ferrous iron transporter *Leishmania* iron transporter 1 (LIT1) (Huynh et al., 2006; Jacques et al., 2010). Moreover, LIT1-mediated iron acquisition seems to be essential for the differentiation of *L. amazonensis* parasites from the sandfly promastigote form to the macrophage-adapted amastigote form (Mittra et al., 2013).

Apart from the mechanisms of direct iron internalization, *Leishmania* parasites can also subvert the host's iron uptake systems to their own advantage. In fact, *L. amazonensis* amastigotes can obtain transferrin by forcing the fusion of transferrin-containing endosomes with the parasitophorous vacuole (Borges et al., 1998). Alternatively, *L. donovani* is capable of decreasing the macrophage labile iron pool, a process that triggers an increased surface expression of transferrin receptor 1 and internalization of transferrin, thus permitting a continuous provision of iron to the parasite (Das et al., 2009). This decrease in labile iron pool of activated macrophages has been recently proposed to be the result of the down-regulation of the expression of SLC11A1 by a *L. donovani*-secreted peroxidase (Singh et al., 2013). Also in line with these data, it has been reported that the expression of ferroportin is down-regulated in the spleen of *L. donovani*-infected mice, which may contribute to an increased accumulation of iron inside macrophages (Yang et al., 2002).

The existence of a parasitic strategy to counteract host SLC11A1 action reinforces the involvement of the latter in the *in vivo* control of infection by *Leishmania* (Vidal et al., 1995; Searle et al., 1998; White et al., 2005), as mentioned previously. This protein has also been implicated in the response to vaccination. Mice with functional SLC11A1 mount primarily a Th1 response to vaccination with the parasite metalloprotease Gp63 and display decreased skin lesions during a challenge infection with *L. major*. In striking contrast, mice with mutated SLC11A1 exhibit a Th2 response and an exacerbated lesion growth upon challenge (Soo et al., 1998).

Finally, we should acknowledge that the involvement of other host mechanisms of iron deprivation during leishmanial infections is largely unknown.

## Iron deprivation vs. iron-induced toxicity

As the number of antibiotic resistant pathogens increases and the discovery of new antibiotics declines, the understanding of critical pathways in host-pathogen interaction emerges as a promising source of new approaches to fight infections. The modulation of iron availability may be one of such pathways. However, it should be noted that the choice of whether to provide or to deprive the pathogen of iron clearly depends on the microorganism in question.

The first widely used treatments for leishmaniasis relied on the administration of antimony complexes. Not excluding other possible mechanisms of action, strong evidences suggest that antimony compounds kill *Leishmania* parasites through the increased generation of ROS in the host (Ait-Oudhia et al., 2011) similarly to what we and others recently described for iron (Bisti et al., 2006; Vale-Costa et al., 2013). Of interest, other metal-containing drugs continue to be described as potential new therapies against *Leishmania* (Vale-Costa et al., 2012; Rocha et al., 2013). The main concern raised by these metal-providing tools is obviously the inherent toxicity of the metals associated with their propensity to induce oxidative stress. One of the possible ways to circumvent this problem will be to use new specific delivery systems to target the drug to infected macrophages.

In the case of mycobacterial infections, it is well-established that iron deprivation inhibits pathogen proliferation and iron depriving strategies seem the most promising in therapeutic terms. However, the administration of iron chelators is not exempt from risks to the host, especially in a context where the latter activates a series of iron withholding mechanisms which may lead to anaemia. The findings of the last decade on the players that control host iron metabolism, notably the identification of the iron exporter ferroportin and the hormone hepcidin, have opened new and exciting possibilities for the modulation of iron availability and localization inside cells which should provide ways of specifically depriving intracellular pathogens, without hampering the normal iron homeostasis of the host.

## Funding

Project “NORTE-07-0124-FEDER-000002-Host-Pathogen Interactions” co-funded by Programa Operacional Regional do Norte (ON.2—O Novo Norte), under the Quadro de Referência Estratégico Nacional (QREN), through the Fundo Europeu de Desenvolvimento Regional (FEDER) and by FCT (Fundação para a Ciência e Tecnologia).

### Conflict of interest statement

The authors declare that the research was conducted in the absence of any commercial or financial relationships that could be construed as a potential conflict of interest.

## Abbreviations

ACD: anaemia of chronic disease
β2m: beta-2-microglobulin
DFO: desferrioxamine
HH: hereditary hemochromatosis
Lcn-2: Lipocalin 2
MHC-I: major histocompatibility complex class I
NO: nitric oxide
NOS2: nitric oxide synthase 2
ROS: reactive oxygen species.
